# Cellular stress response, redox status, and vitagenes in glaucoma: a systemic oxidant disorder linked to Alzheimer’s disease

**DOI:** 10.3389/fphar.2014.00129

**Published:** 2014-06-06

**Authors:** Angela Trovato Salinaro, Carolin Cornelius, Guido Koverech, Angela Koverech, Maria Scuto, Francesca Lodato, Vincenzo Fronte, Vera Muccilli, Michele Reibaldi, Antonio Longo, Maurizio G. Uva, Vittorio Calabrese

**Affiliations:** ^1^Department of Biomedical Sciences, School of Medicine, University of CataniaCatania, Italy; ^2^Department of Chemistry, School of Medicine, University of CataniaCatania, Italy; ^3^Department of Ophthalmology, School of Medicine, University of CataniaCatania, Italy

**Keywords:** free radicals, stress response, vitagenes, hormesis, antioxidants

## Abstract

Amyloid deposits, constituted of amyloid beta (Aβ) aggregates, are a characteristic feature of several neurodegenerative diseases, such as Alzheimer’s, mild cognitive impairment and Parkinson’s disease. They also have been recently implicated in the pathogenesis of retinal damage, as well as age-related macular degeneration and glaucoma. Glaucoma is a progressive optic neuropathy characterized by gradual degeneration of neuronal tissue due to retinal ganglion cell loss, associated to visual field loss over time resulting in irreversible blindness. Accumulation of Aβ characterizes glaucoma as a protein misfolding disease, suggesting a pathogenic role for oxidative stress in the pathogenesis of retinal degenerative damage associated to glaucoma. There is a growing body of evidence demonstrating a link between Alzheimer’s disease and glaucoma. Further, several heat shock proteins (HSPs) members have been implicated both in neurodegenerative diseases and glaucomatous apoptosis. To maintain redox homeostasis vitagenes, as integrated mechanisms, operate actively to preserve cell survival under condition of stress. Vitagenes encode for sirtuin, thioredoxin and HSPs. The present study was designed to investigate cellular stress response mechanisms in the blood of patients with glaucoma, compared to control subjects. Levels of vitagenes HSP-72, heme oxygenase-1, as well as F2-isoprostanes were significantly higher in the blood of patients with glaucoma than in controls. Furthermore, in the same experimental group increased expression of Trx and sirtuin 1 were measured. Our results sustain the importance of redox homeostasis disruption in the pathogenesis of glaucoma and highlights the opportunity that new therapies that prevents neurodegeneration through non-immunomodulatory mechanisms might be synergistically associated with current glaucoma therapies, thus unraveling important targets for novel cytoprotective strategies.

## INTRODUCTION

Glaucoma is a progressive optic neuropathy characterized by degeneration of neuronal tissue due to loss of retinal ganglion cells (RGCs), with accompanying compromission of visual field over time (Gupta et al., 2006; Quigley and Broman, 2006). It is a leading cause of irreversible blindness estimated to affect 79.6 million people worldwide by 2020 (Hinton et al., 1986; Yoneda et al., 2005). Research studies have demonstrated that RGC damage in glaucoma is not limited to the primary insulted neurons, but also involves neighboring neurons. The increase in the prevalence of glaucoma with age is not accounted for only by the increase in ocular hypertension alone, being accompanied by an increase in the vulnerability of the optic nerve to the effects of glaucoma risk factors which increase as function of age. In particular, factors such as tissue hypoxia, disturbed protein metabolism and oxidative stress have been identified to interact in a vicious cycle underlying the pathogenesis of glaucoma (Calandrella et al., 2007; Gupta et al., 2008), ultimately leading to apoptotic retina ganglion cell death (Tatton et al., 2001; Soti et al., 2005; De la Monte and Wands, 2006). In view of these considerations glaucoma can be viewed as a neurodegenerative disease which, similarly to other neurodegenerative pathologies, i.e., Alzheimer’s and Parkinson’s disease, where irreversible functional deficit ensues as consequence of neuronal dysfunction and death. There is now a growing body of evidence demonstrating a link between AD and glaucoma.

Amyloid deposits, consisting of Aβ, which are a characteristic feature of several neurodegenerative diseases such as Alzheimer’s (AD), mild cognitive impairment, and Parkinson’s disease (Hinton et al., 1986) have been recently implicated in the pathogenesis of retinal damage, macular degeneration, and glaucoma (Yoneda et al., 2005). Accordingly, drugs designated to target β-amyloid (Aβ) has been found to reduce apoptotic degeneration of RGCs, as observed *in vitro* and *in vivo*. Furthermore, the presence of increased levels of Aβ characterizes glaucoma as a protein misfolding disease, also suggesting a role for oxidative stress in the pathogenesis of retinal degenerative damage associated to glaucome. Although oxidative stress has been recognized to play a critical role in the development and progression of glaucoma, yet, the exact mechanisms remain elusive. Oxidative stress can cause oxidative attack to DNA, proteins, and lipids, leading to DNA and protein modification, thus sustaining the pathophysiology of degenerative damage of RGCs (Gupta et al., 2006). Relevant to protein misfolding, of emerging interest are heat shock proteins (HSPs), specialized molecular chaperones which mediate various cellular functions. HSPs are up regulated in response to conditions of stress in order to restore normal cell integrity (Soti et al., 2005). The heat shock response, an important component of vitagene family, contributes to establishing a cytoprotective state in a wide variety of human diseases. Vitagenes include, besides HSPs 70 and 32, the latter also called heme oxygenase-1 (HO-1), thioredoxin and sirtuins (Butterfield et al., 2010, 2011). Several families of HSP have been implicated in neurodegenerative diseases and glaucomatous RGC apoptosis with increased levels of circulating autoantibodies to alpha-crystallins and HSP-27 and increased immunostaining of HSP-60, HSP-27 in RGCs and the retinal blood vessels in glaucoma patients (Shieds et al., 1996; Izzotti et al., 2006). In a rat glaucoma model, treatment with geranylgeranylacetone increases HSP-72 synthesis while reducing markedly RGC loss, possibly through interactions with different protein kinases, such as Akt kinase, and the inhibition of NF-kB. In this study we tested the hypothesis that there may be a causal relationship between AD and glaucoma that may be explained by systemic oxidative stress and dysregulation of cellular stress response. Present work elucidated cellular stress response in peripheral cells in patients with glaucoma as compared to healthy volunteers, as control, in order to gain insight into the pathogenic mechanisms operating in the neurodegenerative damage associated with this disease and exploit the possible role of vitagenes in opening up new therapeutic targets for limiting the oxidative damage associated to degeneration occurring in glaucoma.

## MATERIALS AND METHODS

### PATIENTS

Eighteen patients (12 males and six females, mean age 60 ± 15 years) with diagnosis of hypertensive primary open-angle glaucoma (POAG), with typical optic nerve head and visual field damage, were included in the study. Mean MD and PSD were respectively -7.5 ± 8.6 dB, and 4.2 ± 3.8 dB. Twenty age-matched healthy volunteers were recruited as controls. Patients and control subjects underwent IOP measurement by Goldmann applanation tonometer, optic nerve head examination by 78 D lens at the slit lamp, and visual field test 24-2 SITA standard, by a 750 Humprey perimeter (HFA II). Clinical characteristics of patients and control subjects are shown in **Table 1**. Patients with normal tension glaucoma, previous uveitis, diabetes, arterial hypertension were excluded. The study was conducted according to guidelines of local Ethics Committee, and informed consent was obtained from all patients.

**Table 1:** Clinical data of glaucoma patients and control subjects.

	Number of subjects	Age (Mean ± SD)	Gender (F/M)	md (Mean ± SD)	Psd (Mean ± SD)
Patients	18	60 ± 15	7/1	-7.5 ± 8.6	4.2 ± 3.8
Controls	20	73 ± 5	2/8	-1.2 ± 1.1	0.8 ± 0.3

*Md, mean defect; psd, pattern standard deviation.*

### SAMPLING AND LYMPHOCYTE PURIFICATION

Blood (5 ml) collected from controls and patients into tubes containing EDTA, was divided into two aliquots, 1 and 4 ml respectively. One aliquots (1 ml) was centrifuged at 3000 × *g* for 10 min at 4°C to separate serum from red blood cells, while 4 ml aliquots, were utilized for lymphocytes purification, which was accomplished by using the Ficoll Paque System following the procedure provided by the manufacturer (GE Healthcare, Piscataway, NJ, USA).

### WESTERN BLOT ANALYSIS

HSP-70, HO-1, Trx, and Sirt-1 protein levels were estimated by Western blot analysis which was accomplished as previously described in Calabrese et al. (2012). Plasma samples were processed as such, while the isolated lymphocyte pellet was homogenized and centrifuged at 10,000 × *g* for 10 min. The supernatant was then used for analysis after determination of protein content. Proteins extracted for each sample, at equal concentration (40 μg), were boiled for 3 min in sample buffer (containing 40 mM Tris-HCl pH 7.4, 2.5% SDS, 5% 2-mercaptoethanol, 5% glycerol, 0.025 mg/ml of bromophenol blue) and then separated on a polyacrylamide mini gels precasting 4–20% (cod NB10420 NuSept Ltd Australia). Separated proteins were transferred onto nitrocellulose membrane (BIO-RAD, Hercules, CA, USA) in transfer buffer containing (0.05% di SDS, 25 mM di Tris, 192 mM glycine and 20% v/v methanol). The transfer of the proteins on the nitrocellulose membrane was confirmed by staining with Ponceau Red which was then removed by three washes in PBS (phosphate buffered saline) for 5 min each. Membranes were then incubated for 1 h at room temperature in 20 mM Tris pH 7.4, 150 mM NaCl and Tween 20 (TBS-T) containing 2% milk powder and incubated with appropriate primary antibodies, namely anti-HSP-70, anti-HO-1, anti-Trx and anti Sirt-1 polyclonal antibody (Santa Cruz Biotech. Inc.), overnight at 4°C in TBS-T. The same membrane was incubated with a goat polyclonal antibody anti-beta-actin (SC 1615 Santa Cruz Biotech. Inc., Santa Cruz, CA, USA, dilution 1:1000) to verify that the concentration of protein loaded in the gel was the same in each sample. Excess unbound antibodies were removed by three washes are with TBS-T for 5 min. After incubation with primary antibody, the membranes were washed three times for 5 min. in TBS-T and then incubated for 1 h at room temperature with the secondary polyclonal antibody conjugated with horseradish peroxidase (dilution 1:500). The membranes were then washed three times with TBS-T for 5 min. Finally, the membranes were incubated for 3 min with SuperSignal chemiluminiscence detection system kit (Cod 34080 Pierce Chemical Co, Rockford, IL, USA) to display the specific protein bands for each antibody. The immunoreactive bands were quantified by capturing the luminescence signal emitted from the membranes with the Gel Logic 2200 PRO (Bioscience) and analyzed with Molecular Imaging software for the complete analysis of regions of interest for measuring expression ratios. The molecular weight of proteins analyzed was determined using a standard curve prepared with protein molecular weight.

### MEASUREMENT OF F2-ISOPROSTANES

F2-isoprostanes were determined by HPLC according to the procedure of Ritov et al. (2002). F2-isoprostane content in plasma was expressed in nM.

### DETERMINATION OF PROTEIN

Protein concentration in all experimental samples was determined by the bicinchoninic acid protein assay (Pierce, Rockford, IL, USA) according to Smith et al. (1985) and using serum bovine albumin as standard.

### STATISTICAL ANALYSIS

Results were expressed as means ± S.E.M. Each experiment was performed, unless otherwise specified, in triplicate. Data were analyzed by one-way ANOVA, followed by inspection of all differences by Duncan’s new multiple-range test. Differences were considered significant at *P* < 0.05.

## RESULTS

**FIGURE 1:**
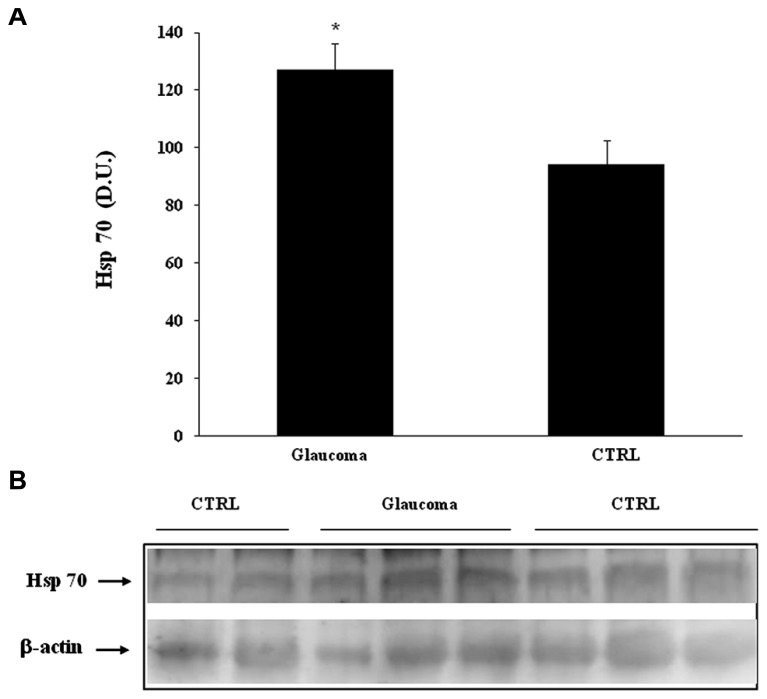
**(A)** HSP-70 protein levels in plasma of glaucoma and control subjects. Samples from control and glaucoma patients were assayed for HSP-70 expression by Western blot. A representativ eimmunoblot is shown. β-actin has been used as loading control. **(B)** Densitometric evaluation: the bar graph shows the values are expressed as mean standard error of mean of 3 independent analyses. *P < 0.05 vs. control. D.U., densitometric units; CTRL, control.

**FIGURE 2:**
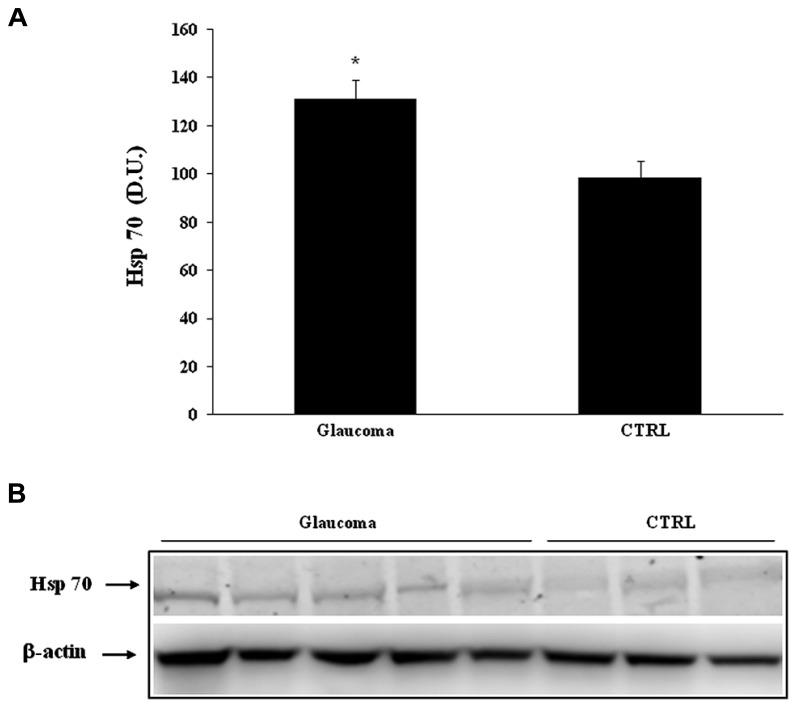
**(A)** HSP-70 protein levels in lymphocytes of glaucoma and control subjects. Samples from controls and glaucoma patients were assayed for HSP-70 expression by Western blot. A representative immunoblot is shown. β-actin has been used as loading control. **(B)** Densitometric evaluation: the bar graph shows the values are expressed as mean standard error of mean of 3 independent analyses. *P < 0.05 vs. control. D.U., densitometric units; CTRL, control.

In our study we evaluated the expression of stress proteins in plasma and lymphocytes of glaucomatous patients compared to controls. Among the 70 kDa family of HSPs we evaluated the inducible HSP-70 (HSP-72) isoform and its expression. As shown in **Figure 1A**, a significant (*P* < 0.05) increase in HSP-70 was found in lymphocytes of patients with glaucoma with respect to healthy control subjects. A representative immunoblot is reported in **Figure 1B**. Western blot analysis of plasma probed for HSP-70 are reported in **Figure 2A**, which shows that HSP-70 expression increased significantly (*P* < 0.05) in patients with glaucoma, compared to controls. A representative immunoblot is shown in **Figure 2B**.

**FIGURE 3:**
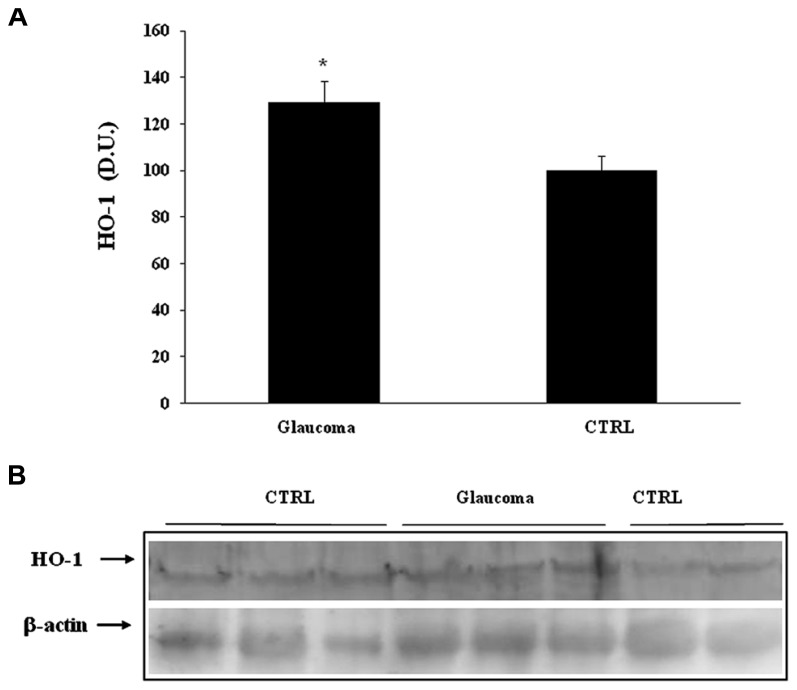
**(A)** HO-1 protein levels in plasma of glaucoma and control subjects. Samples from control and patients with glaucoma were assayed for HO-1 expression by Western blot. A representative immunoblot is shown. β-actin has been used as loading control. **(B)** Densitometric evaluation: the bar graph shows the values are expressed as mean standard error of mean of 3 independent analyses. *P < 0.05 vs. control. D.U., densitometric units; CTRL, control.

**FIGURE 4:**
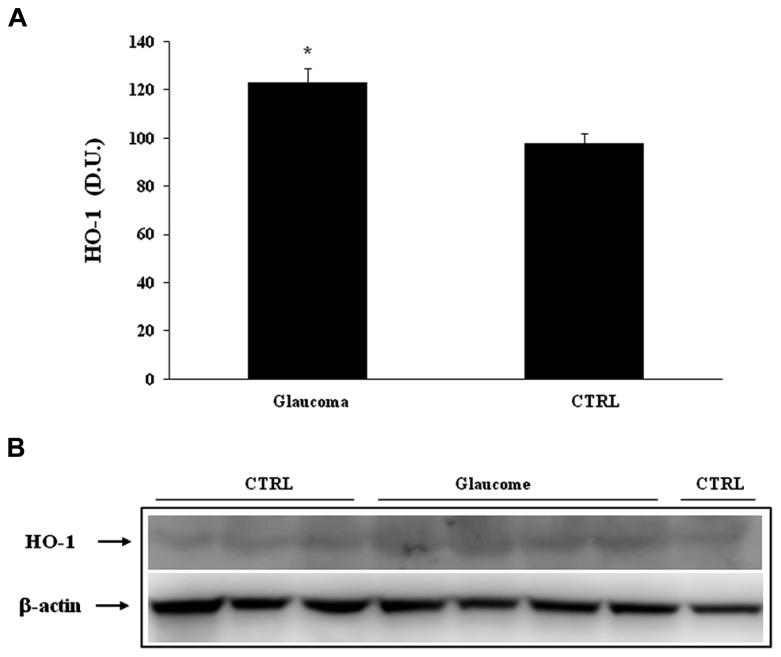
**(A)** HO-1 protein levels in lymphocytes of glaucoma and control subjects. Samples from control and patients with glaucoma were assayed for HO-1 expression by Western blot. A representative immunoblot is shown. β-actin has been used as loading control. **(B)** Densitometric evaluation: the bar graph shows the values are expressed as mean standard error of mean of 3 independent analyses. *P < 0.05 vs. control. D.U., densitometric units; CTRL, control.

Heme oxygenase-1, another HSP-32 endowed with cytoprotective properties (Soti et al., 2005), was found expressed at significantly higher levels in lymphocytes of patients with glaucoma than in controls (**Figure 3A**). A representative immunoblot is illustrated in **Figure 3B**. Similarly to lymphocyte finding, patients with glaucoma exhibited higher level plasma HO-1 protein then healthy controls (**Figures 4A,B**).

**FIGURE 5:**
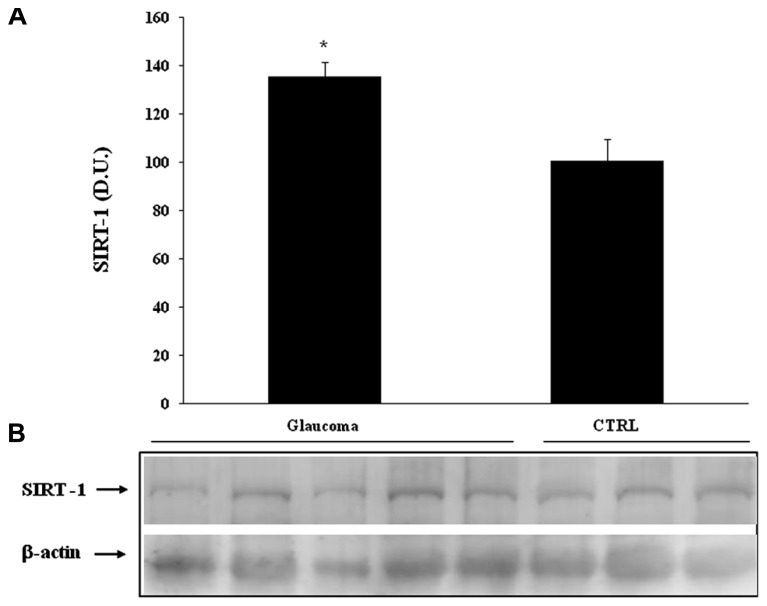
**(A)** Sirtuin-1 protein levels in plasma of glaucoma and control subjects. Samples from control and patients with glaucoma were assayed for Sirt-1 expression by Western blot. A representative immunoblot is shown. β-actin has been used as loading control. **(B)** Densitometric evaluation: the bar graph shows the values are expressed as mean standard error of mean of three independent analyses. *P < 0.05 vs. control. D.U., densitometric units; CTRL, control.

**FIGURE 6:**
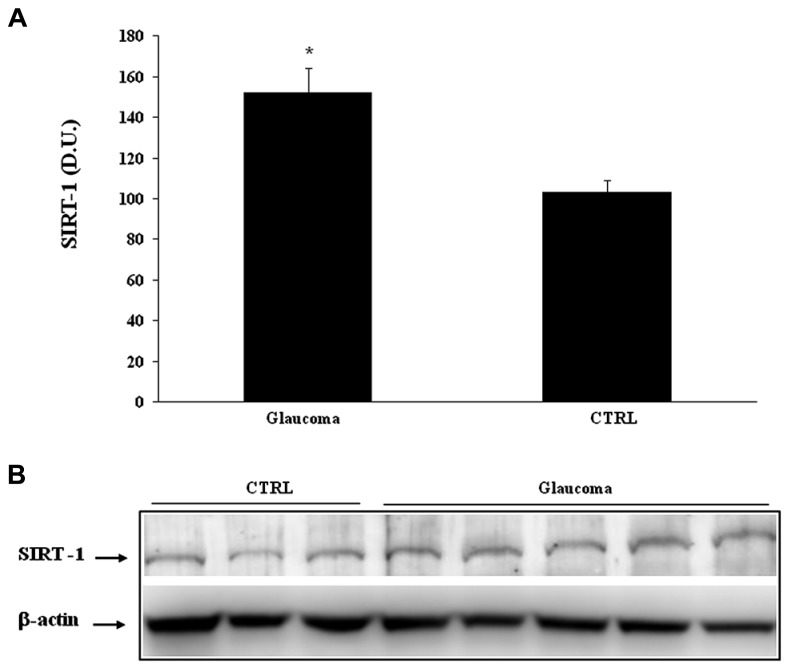
**(A)** Sirtuin-1 protein levels in lymphocytes of glaucoma and control subjects. Samples from control and patients with glaucoma were assayed for Sirt-1 expression by Western blot. A representative immunoblot is shown. β-actin has been used as loading control. **(B)** Densitometric evaluation: the bar graph shows the values are expressed as mean standard error of mean of 3 independent analyses. *P < 0.05 vs. control. D.U., densitometric units; CTRL, control.

Analysis of lymphocytes in patients with glaucoma, compared to control group, revealed a significant (*P* < 0.05) increase of Trx expression (**Figure 5A**), while in the plasma there was no statistically significant difference between the two experimental groups (**Figure 6A**). Representative immunoblots are reported in **Figures 5B and 6B**, respectively.

**FIGURE 7:**
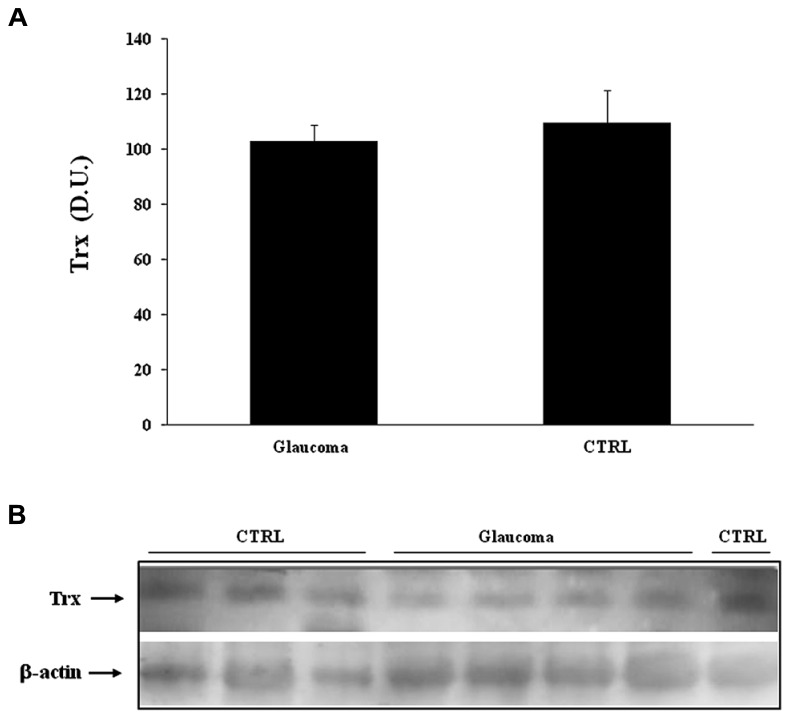
**(A)** Thioredoxin protein levels in plasma of glaucoma and control subjects. Samples from control and patients with glaucoma were assayed for Trx expression by Western blot. A representative immunoblot is shown. β-actin has been used as loading control. **(B)** Densitometric evaluation: the bar graph shows the values are expressed as mean standard error of mean of 3 independent analyses. *P* < 0.05 vs. control. D.U., densitometric units; CTRL, control.

**FIGURE 8:**
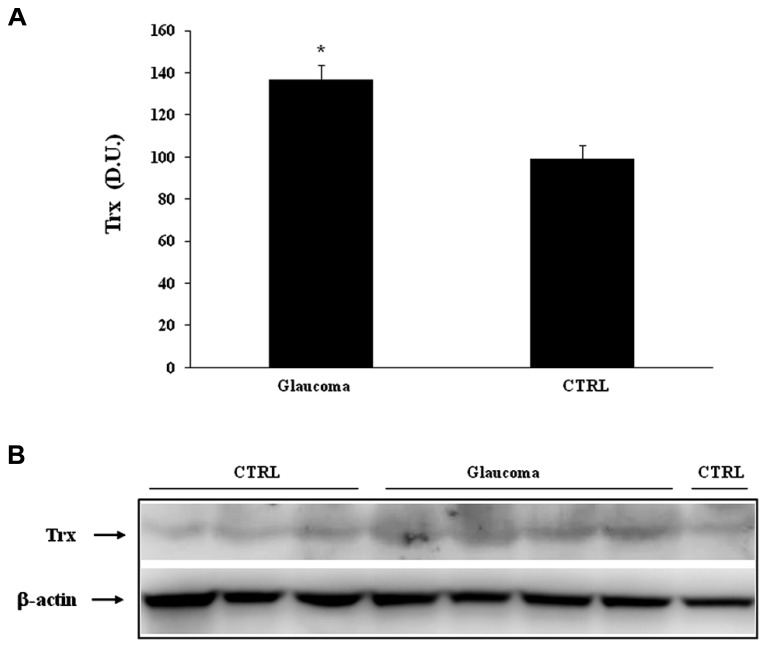
**(A)** Thioredoxin protein levels in lymphocytes of glaucoma and control subjects. Samples from control and patients with glaucoma were assayed for Trx expression by Western blot. A representative immunoblot is shown. β-actin has been used as loading control. **(B)** Densitometric evaluation: the bar graph shows the values are expressed as mean standard error of mean of 3 independent analyses. *P < 0.05 vs. control. D.U., densitometric units; CTRL, control.

Interestingly, we found significantly (*P* < 0.05) higher levels of sirtuin-1 in lymphocytes of patients with glaucoma than in the control group (**Figure 7A**). Consistent with the changes found in lymphocytes, analysis of plasma confirmed increased protein levels of sirtuin-1, higher in patients with glaucoma as to compare with the healthy control group (**Figure 8A**). Representative immunoblots are shown in **Figures 7B and 8B**, respectively. As to our knowledge this is the first evidence of changes in sirtuin-1 expression in glucomatous pathology, although this finding may not be a marker specific for this progressive chronic inflammatory systemic disease.

**FIGURE 9:**
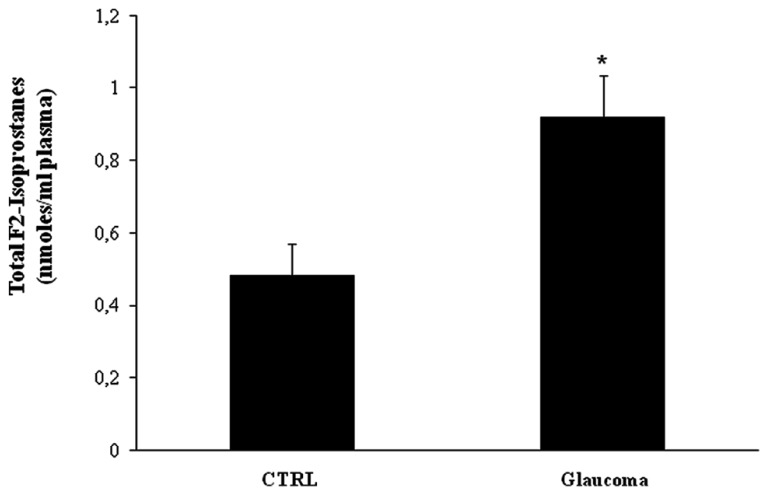
**Total F2-isoprostanes levels in plasma glaucoma patients**. Plasma samples from patients with glaucoma and age-matched controls were assayed for total F2-isoprostanes. Data are expressed as mean ± SEM of 18 to 20 patients per group. *P < 0.05 vs. controls.

Further, we evaluated systemic pro-oxidant conditions, by measuring lipid-derived circulating F2 isoprostanes. We found a significant increase (*P* < 0.05) of total F2-isoprostanes in the plasma of patients with glaucoma (*P* < 0.05) with respect to controls (**Figure 9**).

## DISCUSSION

Glaucoma is one of the leading causes of vision loss worldwide. Open-angle glaucoma, the most common form of glaucoma, is characterized by a progressive loss of RGCs and atrophy of the optic nerve, resulting in loss of visual field (Hinton et al., 1986; Yoneda et al., 2005). Several theories have been proposed, including mechanical and vascular pathogenesis for the glaucomatous optic neuropathy. Elevated intraocular pressure is a strong risk factor, but a subset of glaucoma patients has normal intraocular pressure designating a normal tension glaucoma. Clearly, other factors different from intraocular pressure, such as genetic factors, are thought to be involved in RGC apoptotic cell death in glaucoma. The e4 allele of the APOE gene has been also considered in the pathophysiology of open-angle glaucoma, although the question still remains elusive (Quigley and Broman, 2006).

Oxidative stress is considered an important risk factor for the development of primary angle-closure glaucoma and increased levels of oxidative stress products have been documented in primary angle-closure glaucoma (Shieds et al., 1996; Izzotti et al., 2006; Butterfield et al., 2011). Visual loss which often starts in the periphery and advances involving central vision, has devastating consequences to patient’s quality of life (Gupta et al., 2006; Quigley and Broman, 2006).

We have recently demonstrated that increased oxidative stress and cellular stress response are a systemic presentation of the oxidative burden occurring in AD patients, rising the conceivable possibility that Alzheimer’s disease might not be exclusively a primary neurological pathology rather being a systemic oxidant disorder (Siciliano et al., 2011; Cornelius et al., 2013). In this study we hypothesize that there may be a causal relationship between AD and glaucoma that may be explained by systemic oxidative stress and dysregulation of cellular stress response. We have found in patients with glaucoma a systemic condition of oxidative stress as revealed by upregulation of lipid-derived F2 isoprostanes. This marker of oxidative stress was found in the blood of patients with glaucoma at significantly higher levels than in controls. Similarly to other oxidant disorders, such as AD (Calabrese et al., 2012; Mayeux and Stern, 2012) or multiple sclerosis (Calabrese et al., 2010b) a direct relationship, although not necessarily causal, may exist between organ specific pathology and systemic alterations underlying or reflecting the local oxidative status (Ferreira et al., 2004). Reactive oxygen species (ROS) are an essential component of intracellular signaling network, regulated through the intrinsic antioxidant capacity of a cell, but when ROS formation exceedingly increases damage to DNA, proteins, and lipids macromolecules ensues. During cellular metabolism mitochondrial compartment accounts for the major source of ROS generation. However, excess in free radical production induces oxidative stress and damage. Growing evidence now sustains a critical role for free radical-induced oxidative damage in glaucomatous neurodegeneration occurring in different subcellular compartments of RGCs. Consistent with this notion, oxidatively modified proteins and advanced glycation end products accumulate in glaucomatous neurodegeneration, thus increasing neuronal susceptibility to glial dysfunction (Shieds et al., 1996; Sloane et al., 2002; Tamura et al., 2006). This last event, in turn, contributes to propagate neuronal damage resulting in secondary degenerative damage. Furthermore, free radical-mediated oxidative insult in glaucoma enhances antigen presenting activity of glial cells and hence stimulates immune response (Tezel, 2006; Calandrella et al., 2007).

Oxidative damage is one of the most important causes of brain protein damage and dysfunction in several age-related neurodegenerative disorders including Alzheimer’s disease (Sloane et al., 2002). RGCs and the optic nerve have demonstrated similar mechanisms of cell death in glaucoma to those of Alzheimer’s disease, marking glaucoma as a neurodegenerative disease (Guo et al., 2006; Pennisi et al., 2011). AD is a progressive neurodegenerative disorder characterized by cognitive and memory deterioration, as well as changes in personality, behavioral disturbances and an impaired ability to perform activities of daily living (McKinnon, 2003; Wostyn et al., 2009; Siciliano et al., 2011). AD is known to be the most common form of dementia and is a major public health problem throughout the world (Calabrese et al., 2012). In addition to synaptic degradation and extensive neuronal cell loss, neuropathological characteristics of AD include extracellular senile plaques containing β-amyloid (Aβ) derived from β-amyloid precursor protein (APP) after sequential cleavage by b-secretase and c-secretase, and intracellular neurofibrillary tangles caused by abnormally phosphorylated tau protein (De la Monte and Wands, 2006).

There is a growing body of evidence demonstrating a link between AD and glaucoma. However, the nature of this link remains obscure. Interestingly, recently published research may provide a clue toward a better understanding of the high rate of comorbidity reported between AD and glaucoma.

It is intriguing to note that AD and glaucoma have many common features. Both are slow and chronic neurodegenerative disorders with a strong age-related incidence. Studies consistently report decreased levels of β-amyloid and increased levels of tau in cerebrospinal fluid from AD patients in comparison with healthy subjects. Similarly, decreased levels of β-amyloid and significantly increased levels of tau have been detected in the vitreous fluid from patients with glaucoma or diabetic retinopathy in comparison with the levels in a control group (Calabrese et al., 2010b; Pennisi et al., 2011; Siciliano et al., 2011; Cornelius et al., 2013). This finding corroborates a role for β-amyloid and tau in the pathogenesis of glaucoma, suggesting that the neurodegenerative process in these ocular diseases might share, at least in part, a common mechanism with AD. It was also demonstrated recently that abnormal tau AT8 is present in human glaucomas with uncontrolled elevated intraocular pressure. Furthermore, there is evidence of a build-up of Aβ in RGCs in experimental rat glaucoma. Activation of caspases and abnormal APP processing, which includes production of Aβ are important events in AD (Bullock and Hammond, 2003; Guo et al., 2006).

To gain further insight into the role of oxidant/antioxidant balance in the pathogenesis of glaucoma, in addition to oxidative stress, expression of Sirt-1 and Trx was determined in the peripheral blood of glaucomatous patients. Interestingly, levels of vitagenes HSP-72 and HO-1 were significantly higher in the blood of patients with glaucoma than in controls. These changes were associated with an increased expression of Trx and sirtuin 1 in the same experimental group.

To adapt to environmental changes and survive different types of injuries, as in the case of acute or chronic stress, exposed cells are continually challenged to activate integrated survival responses (Bullock and Hammond, 2003). One of these, the heat shock response actively operate in the optic cell system, under control of redox regulated gene network, the vitagene network, recognized to be critical for the intracellular chaperoning function which is essential for the proper folding of misfolded or mutated proteins, thereby protecting vulnerable cells from death (Selkoe, 2001; Rocchi et al., 2003; Guo et al., 2006). As stress inducible proteins, chaperones help the correct folding and maintenance of the proper conformation of essential proteins, thus promoting cell survival in all those pathological conditions associated oxidative stress (Hirota et al., 2002; Calabrese et al., 2010c). Under oxidative stress conditions, such as that found in patients with glaucoma, HO-1 was also found increased in lymphocytes and plasma of patients with glaucoma. HO-1 is an early gene induced by oxidative stress producing powerful antioxidant and antinitrosative molecules such as biliverdin and bilirubin (Halliwell, 2006; Calabrese et al., 2010a, 2013). HO-1 increase in the lymphocytes of patients with glaucoma may indicate that, in response to an oxidant insult, induction of an early gene is a significant part of the antioxidant response which might have biological relevance considering the long term course of the disease. Under stress conditions, induction of sirtuins is a well recognized defense mechanism against oxidative injury, representing a common feature in a number of neurodegenerative diseases (Salminen et al., 2008). Here we found that the levels of Sirt-1 in glaucoma lymphocytes were significantly higher than in controls, a finding associated with increased content of F2-isoprostanes as marker of oxidative stress. This is relevant to the pathogenesis of glaucoma. Several studies suggest that the Sirt-1 gene is redox-regulated and its expression appears closely related to conditions of oxidative stress (Drake et al., 2003; Bonda et al., 2011). Thus, its induction could represent a protective system potentially active against brain oxidative injury (Kessing et al., 2007; Herranz and Serrano, 2010). In addition, another protein, thioredoxin (Trx), which is emerging as critical vitagene involved in brain stress tolerance was found increased in the same experimental group (Tanaka et al., 2000; Tonissen and Trapani, 2009). Besides its role in the protection against oxidative stress, Trx is critically involved in the regulation of cell growth and cell death (Yi and Luo, 2010; Di Paola et al., 2011). Consistently, modulation of endogenous cellular defense mechanisms such as the vitagene network, including HSPs, sirtuin, and thioredoxin proteins may open a new approaches to therapeutic interventions in diseases associated with tissue damage and cell death, such as in glaucomatous neurodegeneration (Dali-Youcef et al., 2007; Dumont et al., 2009; Ballard et al., 2011). Our data are in favor of the hypothesis linking oxidative stress to the pathogenesis of glaucoma, and indicate that stress responsive genes may represent an important target for novel cytoprotective strategies, as molecules inducing this defense mechanism, via nutritional and/or pharmacological approaches, can exploit the potential for antidegenerative therapeutic interventions.

## Conflict of Interest Statement

The authors declare that the research was conducted in the absence of any commercial or financial relationships that could be construed as a potential conflict of interest.
